# Dengue Fever Resurgence in Iran: An Integrative Review of Causative Factors and Control Strategies

**DOI:** 10.3390/tropicalmed10110309

**Published:** 2025-10-31

**Authors:** Seyed Hassan Nikookar, Saeedeh Hoseini, Omid Dehghan, Mahmoud Fazelidinan, Ahmadali Enayati

**Affiliations:** 1 Health Sciences Research Center, Department of Medical Entomology and Vector Control, School of Public Health, Mazandaran University of Medical Sciences, Sari 48471, Iran; nikookar_84@yahoo.com; 2 MSc Student Research Committee, Department of Medical Entomology and Vector Control, School of Public Health, Mazandaran University of Medical Sciences, Sari 48471, Iran; 3 Tropical and Communicable Diseases Research Centre, Department of Medical Parasitology and Mycology, School of Medicine, Iranshahr University of Medical Sciences, Iranshahr 14155, Iran; omid_dehghan21@yahoo.com; 4 Department of Medical Entomology and Vector Control, Health Sciences Research Center, School of Public Health, Mazandaran University of Medical Sciences, Sari 48471, Iran; fazelidinan@gmail.com; 5 School of Public Health and Health Sciences Research Center, Mazandaran University of Medical Sciences, Sari 48471, Iran

**Keywords:** dengue fever, emergence and re-emergence, *Aedes* mosquitoes, Iran, world

## Abstract

Dengue fever, a mosquito-borne viral disease, has become a significant global health concern in recent decades, particularly in Iran. This integrative review provides a comprehensive assessment of the epidemiological trends of dengue fever in Iran from its initial emergence to the present, identifying and evaluating factors contributing to both the emergence and re-emergence of the disease at national and international levels. The review highlights critical global determinants influencing dengue transmission, including climate change, environmental modifications, unplanned urbanization, increased international travel and trade, and the pivotal roles of public awareness and healthcare infrastructure. Climatic variables, such as rising temperatures and altered precipitation patterns, create favorable conditions for mosquito breeding, enabling vectors like *Aedes aegypti* and *Aedes albopictus* to expand into new regions. We also explore how environmental changes, unplanned urbanization and other factors further exacerbate the situation. Control strategies addressing these factors are presented. In Iran, the incidence of dengue fever is increasing, yet research remains limited. The highest number of local cases has been reported in Chabahar and Bandar Lengeh, Hormozgan and Sistan and Baluchestan Provinces, respectively. Eleven key factors—culminating in socio-cultural practices, climate change, unplanned urbanization, cross-border mobility, and gaps in vector control and healthcare infrastructure—have been identified as contributing to recent outbreaks in Iran. In conclusion, our review underscores that mitigating dengue vulnerability in Iran requires an urgent, multi-faceted strategy targeting improved urban water management, enhanced cross-border surveillance, and strengthened healthcare system capacity.

## 1. Introduction

Recently, due to several fundamental factors, the threat of emerging and re-emerging infectious diseases has been significantly increasing [1], posing a serious threat to human health and wellbeing [2]. These include changes in ecosystems, climate, weather patterns, economic and land use developments, technological and industrial development, population growth, increasing migration rates, and environmental factors [3,4,5].

Dengue fever is one of the most important arboviral diseases in tropical and subtropical regions of the world, transmitted to humans by mosquitoes, particularly *Aedes* species, i.e., *Ae. aegypti* and *Ae. albopictus* [6]. The disease is caused by four serotypes of DENV-1, DENV-2, DENV-3 and DENV-4 of Flaviviridae family, *Orthoflavivirus genus* [7]. These serotypes are antigenically distinct, and infection with one serotype confers lifelong immunity to that specific serotype but only partial and transient protection against the others. Secondary infection with a different serotype increases the risk of severe clinical manifestations, including dengue hemorrhagic fever and dengue shock syndrome [8]. In October 2013, a fifth dengue virus serotype was identified in Sarawak, Malaysia. Unlike the four human-circulating serotypes (DENV-1 to DENV-4), DENV-5 persists in a sylvatic cycle involving non-human primates [8].

About 80% of cases of the disease occur without clinical symptoms or in a mild form. The clinical manifestations of the mild form of the disease include fever, headache, joint and muscle pain, nausea, vomiting, and skin rash [9,10]. In some patients, the appearance of more severe symptoms such as dengue hemorrhagic fever (DHF), dengue shock syndrome (DSS) and multi-organ failure (digestive, liver, kidney, hematological, nervous, respiratory and cardiac) can be life-threatening [10].

Dengue fever is listed as one of the 10 global health threats, in the last two decades [11]. According to the CDC, about 40% of the world’s population lives in areas with a high risk of dengue transmission [12]. Of these, approximately 390 million people are annually infected, 96 million of them show clinical symptoms of any level of severity [13]. Also, most cases of disease and death occur in the age group of 30 to 45 years [14]. The global economic burden of dengue fever is estimated to be up to 306 billion international dollars between 2020 and 2050 [15].

A 10-fold increase in dengue cases (500,000 to 5.2 million) has been reported from 2000 to 2019 [16]. This upward trend was temporarily interrupted in 2020, when cases declined slightly—a shift attributable to COVID-19 pandemic impacts rather than true epidemiological change. Key factors contributing to this decline includes: (1) surveillance disruptions, as overburdened health systems prioritized COVID-19, leading to underreporting of dengue cases [17]; (2) behavioral and mobility changes, including lockdowns, travel restrictions, and reduced outdoor exposure, which limited vector-human contact and imported infections [18]; and (3) diagnostic challenges, where overlapping symptoms (e.g., fever) caused misclassification or delayed dengue confirmation [19]. By 2023, however, cases resurged dramatically, exceeding five million globally, with 5000 deaths across all five WHO regions: Africa, Americas, South-East Asia, Western Pacific, and Eastern Mediterranean [16]. Since the beginning of 2024, more than 12 million dengue cases and more than 8000 dengue deaths have been reported from 86 countries/regions worldwide [20].

The growing trend of global trade, daily movements, global warming and environmental changes contribute significantly to the spread and establishment of invasive mosquito species and the pathogens they transmit across diverse geographical regions. This phenomenon poses substantial threats to public health and local economies [21]. Iran is at risk of *Aedes*-borne diseases because of the presence of *Ae. albopictus* and or *Ae. aegypti* in neighbouring countries including Afghanistan, Armenia, Oman, Pakistan, Saudi Arabia, Turkey and Yemen, and also outbreaks of dengue fever and chikungunya infections in Pakistan, Saudi Arabia, Yemen and Oman [22,23,24,25]. In addition, having a common border with these countries, a large exchange of goods and passengers, an international airport and active commercial ports and transportation routes for goods transit may pave the way for entry, their further distribution and local transmission of the diseases, which the recent presence of *Ae. aegypti* [22,26] and *Ae. albopictus* in Iran [25] and autochthonous dengue cases in Bandar-Lengeh, Hormozgan Province and Chabahar County, Sistan and Baluchistan Province, southern Iran [27] confirms.

*Aedes aegypti* and *Ae. albopictus* are known as the main vectors of dengue worldwide [28]. *Aedes aegypti* is a domestic species originating from Africa [29]. The species was active in southern regions of Iran in the past years (1920 to 1951) [30,31]; however, it likely disappeared subsequently due to the malaria eradication program initiated in 1957 [32]. Recently, the species has been sighted in Hormozgan, Sistan and Baluchistan, and Bushehr Provinces, southern Iran [22,27], raising concerns among health authorities in the country.

*Aedes albopictus* is native to the forests of Southeast Asia [33]. This species was first observed in Sistan and Baluchestan Province on the border with Pakistan in 2009 and in a coastal area near Chabahar county in the same province in 2013 [34]. Despite ongoing entomological surveillance efforts, the establishment of this species in the southern provinces of the country was not confirmed in the following years. Recently, this species has been observed in Gilan Province, northwest of Iran [25], highlighting the necessity for national awareness to prevent and control the spread of this species to other regions

Although there are two vaccines against dengue, they are not in a position to control the diseases universally; therefore, a better understanding of the factors affecting the emergence and re-emergence of dengue is an important step towards finding ways to manage, control, develop preventive policies and strategies, and help health authorities to reduce the impact of the disease on communities. This narrative review provides a comprehensive assessment of the epidemiological trends of dengue fever in Iran, from its initial emergence to the present. It identifies and evaluates factors contributing to both the emergence and re-emergence of dengue at national and international levels. The review also examines relevant control strategies to address these factors.

## 2. Materials and Methods

**Figure 1:**
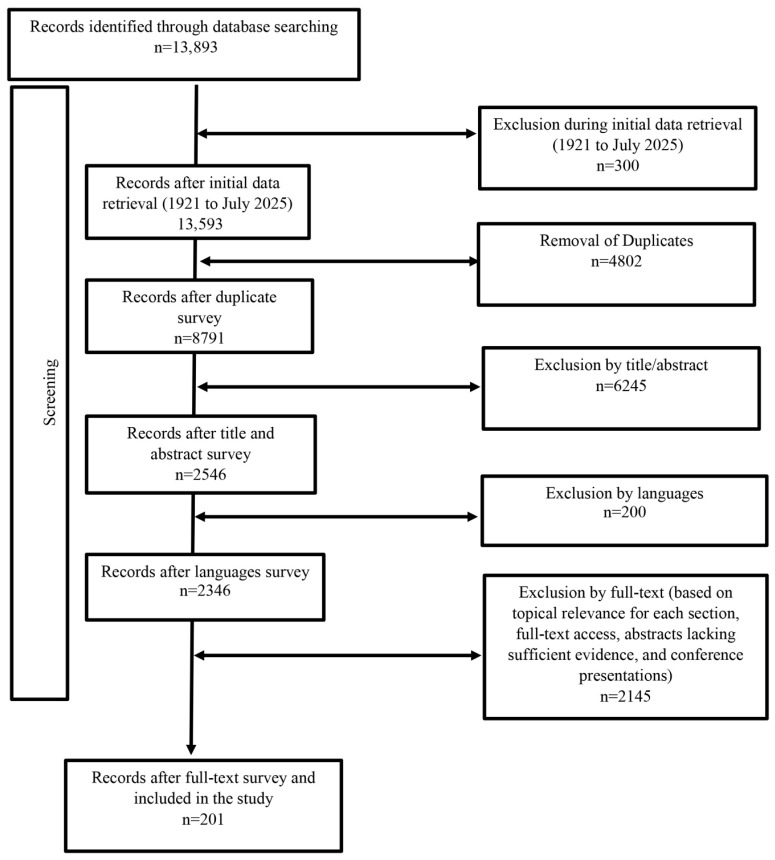
Shows the flow chart of the search strategy, screening, exclusion, and final selection of articles for this review study.

This study employed an integrative narrative review methodology to synthesize diverse evidence from epidemiological, entomological, climatic, and socio-cultural disciplines. This approach was selected to comprehensively map the complex, multifactorial drivers of dengue emergence in Iran, acknowledging that while less systematic than a systematic review and potentially susceptible to selection bias, it provides the necessary flexibility for this exploratory scope. Data collection was conducted through comprehensive searches of international scientific databases, including PubMed, Web of Science, Scopus, and ScienceDirect, as well as Persian databases such as the Scientific Information Database (SID), Magiran, and Civilica. The search utilized both English and Persian keywords, including “Dengue fever”, “emergence”, “re-emergence”, “outbreak”, “control strategies”, “epidemiology”, “*Aedes aegypti*”, “*Aedes albopictus*”, “risk factors”, “global”, and “Iran”. A combination of individual and advanced Boolean search strategies (using AND, OR, NOT) was applied, covering publications from 1921 to July 2025. A sample PubMed search string is provided in Appendix A to enhance reproducibility. Studies were included if they addressed the epidemiology of dengue fever in Iran or examined factors influencing its emergence and re-emergence globally. Articles in languages other than English or Persian were excluded. Following the initial data retrieval, studies were screened for thematic relevance and scientific validity. “Thematic relevance” was defined as alignment with the study objectives, focusing on dengue epidemiology and associated risk factors, while “scientific validity” was assessed based on peer-reviewed publication status and methodological clarity. Official reports from the Iran Center for Disease Control were also considered in our search. Subsequently, relevant data were extracted, and pertinent findings were synthesized using a descriptive-analytical approach. It should be noted that, given the narrative review design and the heterogeneous nature of the included sources (which ranged from original research to reports and reviews), a formal quality assessment of individual studies was not performed. This is acknowledged as a limitation of the chosen methodology. The flowchart illustrating the process of searching, removal, and selection of articles is shown in Figure 1.

## 3. Results

### 3.1. Dengue Fever in Iran

The first confirmed dengue case in Iran was reported in 2008 in a 58-year-old woman with travel history to Malaysia [35]. Subsequent retrospective analyses identified 15 additional probable cases among patients initially suspected of having Crimean-Congo hemorrhagic fever (CCHF), detected through a combination of IgM enzyme-linked immunosorbent assay (ELISA) serology and RT-PCR testing (using CDC DENV-1-4 primers, QIAGEN, Hilden, Germany). These cases showed epidemiological links to travel in Southeast Asia or residence in Sistan and Baluchestan Province—a region climatically suitable for *Aedes* mosquitoes [36]. A study conducted in 2013 provided crucial serological evidence of endemic dengue virus circulation through a study of 540 blood donors in Chabahar. Initial screening by ELISA indicated a 7.6% seropositivity rate; however, subsequent confirmation with immunofluorescence assay (IFA) established the true seroprevalence at 5.9% (32/540). This two-step process revealed that 78% of ELISA-positive samples were confirmed by IFA, highlighting both the extent of asymptomatic infection and the necessity of confirmatory testing. Critically, none of the IFA-confirmed seropositive donors reported a history of international travel [37]. Between 2015 and 2016, two additional travel-associated dengue cases were detected: one in a patient returning from India (IgM+/RT-PCR+) and another from Malaysia (IgM+ only) [38,39]. A pivotal epidemiological shift was observed in 2018, when 60 patients in Zahedan were evaluated. Among them, 13 demonstrated evidence of past or recent dengue virus (DENV) exposure. Serological analysis using ELISA revealed IgM positivity in 6.5% (4/60), IgG positivity in 5% (3/60), and NS1 antigen positivity in 5% (3/60) of patients. Notably, one patient was concurrently positive for IgM and NS1 antigen, and another exhibited both IgG and NS1 antigen without detectable IgM. None of the patients had a history of international travel, suggesting the likelihood of local transmission cycle [40]. This pattern was further confirmed by Tavakoli et al. in a seroepidemiological study of measles/rubella IgM-negative patients presenting with fever and rash, where 82 of 1306 tested sera were positive for dengue infection using IgM antibody detection via ELISA [39].

**Figure 2:**
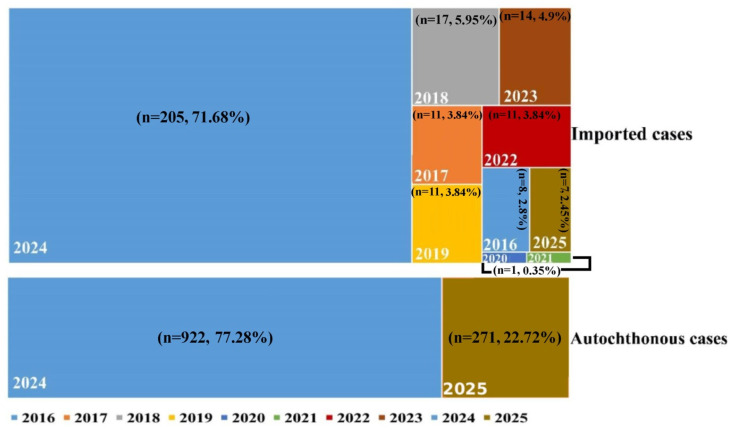
Annual variation in dengue cases in Iran from 2016 to mid-2025. Each block represents a single year, shown in different colors, and the size of the block is proportional to the number of confirmed dengue cases.

According to Iran’s syndromic surveillance system, from 2016 to March 2023, only 74 confirmed dengue cases were reported, all imported from endemic countries (Figure 2 and Table 1). From June to March 2024, a total of 205 imported dengue cases were reported, predominantly originating from neighboring countries and regional travel hubs, including the United Arab Emirates, Pakistan, Oman, Afghanistan, Turkey, and Benin. However, the epidemiological situation changed dramatically in summer 2024, local transmission gradually intensified, so that by the end of March of same year, out of 1127 confirmed cases, 922 locally acquired infections predominantly in Chabahar (Sistan and Baluchestan) and Bandar Lengeh (Hormozgan) (Table 1). Most recently, from April to 19 July 2025, an additional 278 dengue cases have been identified nationwide, continuing this concerning trend (Figure 2 and Table 1) [27].

Surveillance of autochthonous dengue cases in Iran (March 2024–June 2025) revealed hospitalization rates of 6% in the whole of 2024, declining to 2% by mid-2025, with 9% of cases exhibiting warning signs; accordingly, the vast majority of confirmed patients—94% in 2024 and 98% by mid-2025—were managed on an outpatient basis without hospitalization. The most frequent clinical manifestations included fever (100%), headache (82%), myalgia/arthralgia (75%), retro-orbital pain (63%), and calf muscle pain (58%). During this period, dengue diagnosis was performed using a combination of serological methods such as ELISA for dengue-specific IgM and IgG antibodies, NS1 antigen tests for viral non-structural antigen detection, and molecular techniques including reverse transcription polymerase chain reaction (RT-PCR). Among both imported and locally acquired cases, circulation of two dengue virus serotypes, type 1 and type 2, was reported (Personal communications, Iranian Center for Infectious Disease Management (ICDC)).

### 3.2. Key Factors Contributing to the Prevalence of Dengue in World and Their Implications for Iran

#### 3.2.1. Climate Factors

Climate change is expected to expand the geographical range of various *Aedes* species and the diseases they carry, particularly dengue fever [41]. The World Health Organization announced that health issues relevant to climate change are the most important challenge of the 21st century, with dengue fever at the top of their concerns [42]. Temperature, rainfall and humidity are known as climatic factors affecting the dynamics of dengue fever [43]. There is much evidence indicating that dengue epidemics are correlated with temperature [44,45,46], rainfall [47,48], and relative humidity [49,50]. In this review, the effect of each of these factors is discussed separately.

##### Temperature

The forecasts display the mean global temperature will increase between 1.8 °C (low scenario) and 4.0 °C (high scenario) by the end of the 21st century [51]. However, mosquitoes may benefit from a warmer climate, as temperature plays a critical role in the growth, survival, distribution, reproductive activity and characteristics of adult *Aedes* mosquitoes [52]. The optimal temperature range for mosquito growth is 25–30 °C, when it exceeds 40 °C, adults die and eggs and larvae do not develop. Both *Ae. albopictus* and *Ae. aegypti* larvae are unable to grow at 10 °C [53]. A positive correlation has been recorded between *Ae. aegypti* population and minimum temperature, that is, areas with a minimum temperature above 8 °C are more favorable for the survival of the species. The mosquito population increases between the minimum temperatures of 16–20 °C, when it exceeds 20 °C, the population is not affected by further temperature changes [42]. It was shown that *Ae. albopictus* larvae and adults can develop and survive in a wider range of temperatures (10.4–35 °C) [54]. The fact is that surprising results were obtained for this species in various studies, which point to a larval growth temperature threshold of about 9 °C [54,55,56].

Temperature changes affect the external traits of invasive *Aedes* species during the developmental stages. Higher temperatures (24–29 °C with sufficient food) can lead to shorter wings and heavier weight, and conversely, lower temperatures (14–19 °C with insufficient food) can result in mosquitoes with longer wings and less weight [53,57]. The optimum flight temperature of the invasive *Aedes* species, in terms of flight duration and distance, which can affect their behavior including host-seeking, was considered to be 21 °C, but in general, *Ae. aegypti* females have better flight performance than *Ae. albopictus* below 27 °C. Maximum flight speed (34.1 m/min) was also documented at 32 °C and 50% humidity. Reinhold et al. stated that the ability to fly at lower temperatures increases the species’ adaptation to activity during cooler hours of the day (e.g., early morning and late afternoon) [54]. Moreover, as the temperature increases, the extrinsic incubation period (EIP) of *Ae. aegypti* and *Ae. albopictus* decreases to a limit [58]. Temperature have shown a great effect on interaction between host–parasite and vector [59].

**Implication for Iran:** Iran’s significant warming trend, with a projected temperature increase of 1.5–4.0 °C by 2050 [60], will profoundly affect dengue transmission dynamics. Southern provinces like Hormozgan and Sistan & Baluchestan will experience extended seasonal windows for vector activity and accelerated viral replication within mosquitoes due to prolonged heat. Crucially, central and northern regions, including Tehran, may become newly climatically suitable for *Ae. albopictus* establishment [61,62], thereby expanding the geographic risk of dengue beyond the current southern foci and necessitating expanded surveillance and preparedness in these previously unaffected areas.

##### Rainfall

Rainfall is an important factor contributing to dengue incidence in the world [63]. The relationship between rainfall and dengue incidence is complex and multifaceted, involving both direct and indirect mechanisms that affect the lifecycle of the *Aedes* mosquito vectors and the dengue virus itself [63]. Rainfall directly contributes to the availability of breeding sites for *Aedes* mosquitoes. Standing water from rainfall events provides ideal conditions for invasive *Aedes* mosquitoes to lay their eggs, which subsequently develop into larvae and pupae before emerging as adult [64]. This increases their population density, which can lead to a higher risk of dengue transmission when infected by a viremic patient. Moreover, excessive rainfall can also disrupt the invasive *Aedes* mosquitoes breeding cycle through a process known as “flushing” [65]. Flushing occurs when heavy rains wash away the aquatic stages of mosquitoes from their breeding sites, thereby reducing the number of mosquitoes that can potentially transmit the disease [65]. Statistical modeling studies have demonstrated a significant reduction in dengue outbreak risk following flushing events, with a lag time that aligns with the mosquito development cycle and virus transmission period [66]. Indirectly, rainfall influences the behavior of human populations, which can affect the spread of dengue. During rainy seasons, people are more likely to spend time indoors, potentially increasing the proximity between humans and indoor-breeding *Aedes*, i.e., *Ae. aegypti* [41]. Additionally, water storage practices in response to variable rainfall can lead to the creation of additional mosquito breeding sites, particularly in areas with limited access to piped water [66].

The effects of climate change on rainfall patterns, including changes in rainfall distribution and intensity, are likely to have significant implications for dengue transmission. Research indicates that climate variables, such as temperature and rainfall, have nonlinear effects on dengue incidence [67]. For example, minimum temperatures above 18 °C and maximum temperatures around 32 °C, combined with rainfall levels up to approximately 550 mm, are associated with increased dengue incidence [67]. These findings highlight the need for flexible statistical models to capture the complex interactions between climatic factors and dengue transmission.

**Implication for Iran:** In Iran’s arid climate, transmission is primarily driven by human water storage practices rather than rainfall. However, intense, sporadic rainfall events—predicted to increase under climate change—will significantly exacerbate the problem in southern provinces such as Hormozgan, Sistan & Baluchestan, and Bushehr, where inadequate drainage systems are prevalent. These rainfall events create temporary breeding sites in urban areas with poor drainage infrastructure, directly linking climate-induced weather extremes to larval habitat creation [22,68].

##### Humidity

Relative humidity is another key factor that affects the life cycle of mosquitoes at different stages and consequently the prevalence of dengue fever. Increased humidity contributes to the persistence of breeding sites, the survival of adult mosquitoes, and allows them to live longer and transmit the virus more effectively [69]. Higher humidity levels support viral replication within the mosquito’s body, increasing the likelihood of transmission to humans. Additionally, humidity affects mosquito behavior. Mosquitoes tend to be more active during warm and humid weather, increasing their chances of encountering and biting humans. Consequently, this leads to a higher transmission rate of the virus [70]. The minimum humidity for the survival of *Ae. aegypti* is reported to be 60% [71]. Polwiang [72] reported that average humidity of 72.9% played an important role in dengue outbreaks in Bangkok, Thailand. Dickerson (2007) showed a significant positive correlation between increasing relative humidity and reproductive behavior of invasive *Aedes* species [73]. Ebi and Nealon (2016) observed that severe dengue fever cases (1983–2001) peaked when temperatures ranged from 29 to 27 °C and humidity levels exceeded 75% [74]. Understanding the complex interactions between temperature, rainfall, and humidity is crucial for predicting how changing weather patterns can affect dengue fever transmission. Table 2 presents an overview of studies focusing on the influence of climatic factors on the prevalence of dengue fever.

**Implication for Iran:** High humidity on the southern coastlines is a key enabling factor for vector survival and longevity, sustaining transmission in provinces like Hormozgan. In contrast, low humidity inland acts as a major natural barrier to the geographical expansion of vectors into the arid central plateau, currently limiting the risk to coastal and southern regions [75].

**Critical Synthesis:** The authors observed distinct geographical patterns in climate-dengue dynamics. The global evidence demonstrates moderate to strong consistency for temperature and humidity effects on dengue transmission, while rainfall patterns show context-dependent impacts. Specifically, temperature maintains consistent effects on mosquito biology and virus replication across regions, with optimal ranges (25–32 °C) accelerating transmission, while humidity exhibits synergistic enhancement effects with temperature across multiple studied regions. In contrast, rainfall impacts demonstrate significant regional variation, heavily influenced by water storage practices in arid regions versus natural breeding in tropical areas. This geographical heterogeneity underscores that while temperature and humidity provide valuable universal parameters, effective risk assessment requires models adapted to local hydrological conditions and socioeconomic factors (Table 2).

**Table 2:** A summary of the studies conducted on the effect of climate factors on the prevalence of dengue fever, 2001 to 2024.

Author/Year	Country	Climatic Factors Analyzed	Reference
Bi et al. (2001)	Australia	Monthly mean maximum and minimum temperatures, total amounts of precipitation and relative humidity	[76]
Bin-Tang et al. (2003)	China	Average air temperature, lowest air temperature, highest air temperature, sunlight, rainfall and relative humidity	[77]
Thammapalo et al. (2005)	Thailand	Rainfall, Temperature and relative humidity,	[78]
Bangs et al. (2006)	Indonesia	Monthly mean rainfall and temperature	[79]
Wu et al. (2007)	Taiwan	Monthly temperature changes, relative humidity	[80]
Arcari et al. (2007)	Indonesia	Temperature and rainfall	[81]
Su et al. (2008)	Philippines	Rainfall	[82]
Brunkard et al. (2008)	Mexico	Temperature, precipitation, sea surface temperature	[83]
Hii et al. (2009)	Singapore	Average temperature, rainfall	[84]
Lu et al. (2009)	china	Minimum temperature, minimum humidity, wind speed	[85]
Tipayamongkholgul et al. (2009)	Thailand	El Nino phenomenon	[86]
Chen et al. (2010)	Taiwan	Minimum temperature, precipitation, relative humidity	[87]
Pinto et al. (2011)	Singapore	Minimum and maximum temperature	[88]
Gharbi et al. (2011)	Guadeloupe	Relative humidity, average temperature, minimum temperature	[50]
Chowell et al. (2011)	Peru	Average temperature	[89]
Descloux et al. (2012)	Australia	Temperature, relative humidity, rainfall	[45]
Tosepu et al. (2018)	Indonesia	Temperature, rainfall and humidity	[90]
Chang et al. (2018)	Taiwan	Temperature, precipitation and relative humidity	[91]
José et al. (2019)	Brazil	Rainfall and air temperature	[92]
Stolerman et al. (2019)	Brazil	Average rainfall and temperature	[93]
Ye and Moreno-Madriñán (2020)	Columbia	rainfall	[94]
Akter et al. (2020)	Australia	Minimum temperature, maximum temperature and precipitation	[95]
Tran et al. (2020)	Taiwan	temperature	[96]
Shabbir et al. (2020)	Pakistan	Minimum temperature, maximum temperature and average rainfall	[97]
Islam et al. (2021)	Bangladesh	Temperature, precipitation and relative humidity	[98]
Susilawaty et al. (2021)	Indonesia	Temperature, humidity, rainfall and wind speed	[99]
Edussuriya et al. (2021)	Sri Lanka	Average rainfall, humidity, wind speed and temperature	[100]
Polwiang (2021)	Thailand	Rainfall and humidity	[72]
Wang et al. (2022)	Singapore, Malaysia, Sri Lanka, Thailand	Temperature and rainfall	[101]
Singh et al. (2022)	Malaysia	Temperature, wind speed and rainfall	[101]
Hamidun et al. (2022)	Malaysia	Temperature, relative humidity and rainfall	[102]
Pinontoan et al. (2022)	Indonesia	Temperature, rainfall and humidity	[103]
Abdulsalam et al. (2022)	Thailand	Temperature, relative humidity, precipitation, wind speed, evaporation, cloud cover and sea level pressure	[104]
Abdullah et al. (2022)	Malaysia	Temperature, Humidity, Rainfall	[105]
Hossain et al. (2023)	Bangladesh	Mean of maximum and minimum temperature, wind speed, sunshine hour, and rainfall	[106]
Mia et al. (2024)	Bangladesh	Temperature, Humidity, Precipitation, Air Pressure	[107]
Ouédraogo et al. (2025)	West Africa	Mean relative humidity, minimum and maximum temperature, rainfall and wind speed	[108]

#### 3.2.2. Environmental Factors

##### Vegetation

Both natural vegetation (forests, green spaces) and urban greenery (parks, gardens) affect dengue transmission. Urban green spaces can serve as refuges for invasive *Aedes* species or can be a proxy for the presence of breeding and feeding sites [109], while well-maintained natural vegetation may reduce their abundance [110]. Various studies revealed a combination of positive, negative, and nonlinear relationships between vegetation and dengue (Table 3) [111,112,113,114]. These contradictory findings can be attributed to analytical approaches, spatial and temporal scale, and vegetation measurement. Other studies have shown that vegetation management, including planting specific species [114] and optimizing planting configurations [115], can reduce mosquito populations. Therefore, understanding the relationship between vegetation and dengue transmission is essential for effective public health interventions. By managing vegetation strategically, specifically in urban areas with high population density, where other interventions such as land use control and housing improvement may be more challenging and costly, can mitigate the risk of dengue outbreaks.

**Implication for Iran:** The relationship between vegetation and dengue risk in Iran is complex and region-specific. In the north, lush vegetation in provinces like Gilan may provide resting habitats for the recently detected *Ae. albopictus* [116]. In contrast, in arid southern cities, urban greening efforts (e.g., parks and gardens) that require frequent irrigation can inadvertently create persistent larval habitats if water management is poor. The loss of natural vegetation to urban expansion also contributes to the urban heat island effect, which can further elevate temperatures and enhance vector competence in urban centers like Bandar Abbas and Chabahar [26,27].

**Table 3:** Studies on the relationship between vegetation and dengue transmission.

Author/Year	Country	Findings	Relationship Type	Reference
Higa et al. (2010)	Vietnam	Reported that urbanization and loss of vegetation were linked to increased dengue cases, indicating a positive correlation between reduced green spaces and disease transmission.	Positive	[117]
Sarfaraz et al. (2012)	Thailand	Identified significant positive correlations between dengue indices and various land-use types, including deciduous forests and horticultural land.	Positive	[118]
Cheong et al. (2014)	Malaysia	Highlighted the influence of land use, including water bodies and agricultural practices, on dengue cases, indicating complex interactions with vegetation.	Nonlinear	[119]
Pereira da Silva et al. (2022)	Brazil (Cerrado)	Reported a significant relationship between the loss of native vegetation and increased dengue cases, suggesting that deforestation may enhance disease risk.	Positive	[120]
Tewari et al. (2023)	Singapore	Found a strong negative association between forest cover and dengue incidence, suggesting that higher vegetation cover may provide a protective barrier against mosquito populations.	Negative	[121]

##### Deforestation

Deforestation, i.e., the conversion of forest to another use, facilitates the transmission of some infectious diseases including dengue by affecting the ecology of the vector and their habitats [122]. Deforestation has a profound impact on the breeding, population dynamics, and species diversity of mosquito populations [123]. Deforestation generally leads to an increase in the populations of *Aedes* mosquitoes, which are key vectors for various human pathogens, including those responsible for diseases such as dengue fever. Studies have shown that the alteration of natural habitats due to deforestation creates more favorable breeding conditions for these mosquitoes. For instance, the removal of forest cover often results in the formation of stagnant water bodies, which are ideal for mosquito larvae [124]. Furthermore, the loss of canopy cover allows sunlight to penetrate previously shaded areas, promoting the growth of aquatic vegetation and algae that can support mosquito breeding [125]. This ecological shift tends to favor *Aedes* species, leading to increased abundance and, consequently, a higher risk of disease transmission to humans [124]. However, it is important to note that the specific impact of deforestation on mosquito populations can vary based on local environmental conditions and the species involved. While many studies indicate that deforestation enhances the populations of invasive *Aedes* mosquitoes (Table 4), the overall effects may differ depending on the ecological context and the presence of other competing species [123]. Understanding these dynamics is crucial for developing effective public health strategies aimed at controlling vector-borne diseases in deforested regions.

**Implication for Iran:** Deforestation and rapid land-use changes, particularly through villa construction in northern provinces like Gilan and Mazandaran, are creating favorable ecological conditions for the expansion of *Ae. albopictus* into new habitats [116]. Forest loss reduces shade coverage, elevates surface water temperatures, and creates artificial breeding sites through soil erosion and water accumulation in disturbed areas. These modifications not only facilitate the northward expansion of dengue transmission risk into previously unaffected regions but also fragment natural ecosystems, potentially reducing predator diversity that would otherwise regulate mosquito populations. This synergy between habitat disturbance and vector adaptation demonstrates how regional development can mimic the vector-enhancing effects observed globally [126,127], underscoring the critical need to integrate environmental conservation into Iran’s dengue prevention strategies, particularly in ecologically sensitive zones experiencing rapid transformation.

**Table 4:** Summary of studies on the role of deforestation in the emergence of dengue fever, 2000 to 2025.

Author	Country	Purpose of the Study	Findings	Reference
Patz et al. (2000)	America	Investigating the effects of environmental changes on emerging parasitic diseases	Deforestation and land use change disrupt the natural ecosystem and increase the risk of dengue transmission in the human population.	[128]
Vora (2008)	America	Investigating the impact of human environmental changes on vector-borne diseases	Deforestation can change the entire ecosystem of an area. This, in turn, can affect the transmission of vector-borne diseases such as dengue fever by changing the vegetation cover.	[129]
Troyo et al. (2009)	Costa Rica	Investigating the urban and ecological structure and incidence of dengue fever in the city of Puntarenas in Costa Rica	Areas with little vegetation increase the incidence of dengue fever.	[130]
Araujo et al. (2015)	Brazil	Investigating the relationship between heat characteristics and the incidence of dengue fever in Sao Paulo, Brazil during a two-year period (2010–2011) and identifying related factors	In areas with little vegetation, the air temperature was higher than in areas with denser vegetation, and the incidence rate of dengue fever was also higher.	[111]
Husnina et al. (2019)	Indonesia	Description of dengue fever in Sumatra and Kalimantan islands and its relationship with forest cover	The results showed that the risk of dengue fever decreased by 9% with a 1% increase in forest cover.	[131]
Kalbus et al. (2019)	Costa Rica	Investigating the relationship between the incidence of dengue fever and other environmental factors such as deforestation and forest cover in Costa Rica	Changes in environmental factors such as deforestation can increase the distribution of dengue fever.	[132]
Kalbus et al. (2021)	Brazil	Investigating the potential causes of the emergence of dengue fever in the Brazilian Amazon with a focus on deforestation	Deforestation facilitates the emergence of dengue. However, no significant dose–response relationship was found between dengue incidence and deforestation in the Brazilian state of Amazonas in this study.	[133]
Karuppusamy et al. (2021)	India	Assessing the impact of climate change and deforestation on vector-borne diseases and observing their relationship with the epidemiology of dengue fever and malaria	Increasing rainfall, humidity and deforestation can be important factors for the spread of two diseases, malaria and dengue fever.	[134]
Cunha et al. (2021)	Brazil	Investigating the relationship between dengue incidence and vegetation in Brazil during the 2010 dengue epidemic	The results of the study show the potential of vegetation management in reducing the incidence of dengue fever, especially in socially and economically vulnerable areas.	[135]
Da Silva et al. (2023)		Conducting a spatial analysis of the conditioning factors for the increase in the incidence rate of dengue cases in municipalities located in the Amazon biome, in the period from 2016 to 202	The results revealed that the incidence rates of dengue cases are associated with deforestation.	[136]
Piaggio et al. (2024)		Estimating the marginal effects of increasing forest cover on dengue prevalence in Costa Rica using econometric models to relate hospital admission records to forest cover maps from 2001 and 2011.	The findings of the study indicate that an increase in forest cover is significantly associated with a reduction in both dengue-related hospital admissions and the likelihood of outbreaks. The analysis predicts that if forest cover had increased by three percent over a decade (approximately 0.29% annually), around 29 annual hospital admissions for dengue could have been averted (around 1.4% of cases in the country, depending on the year).	[137]
Nawaz and Charles (2025)		Investigation of the effects of deforestation, agricultural expansion, and urbanization on mosquito populations and the dynamics of disease transmission such as dengue fever and Zika virus	The study demonstrated that deforestation alters local ecosystems by disrupting predator-prey relationships, increasing sun exposure, and creating stagnant water bodies that serve as mosquito breeding sites, thereby facilitating conditions conducive to the expansion of dengue fever.	[138]

#### 3.2.3. Expansion of Urbanization

Today, the unprecedented growth of the population, which has been the main driving force of unplanned and uncontrolled urbanization, especially in tropical developing countries, has significantly contributed to the emergence and spread of vector-borne diseases, particularly dengue fever [139]. A study conducted by Qi et al. indicated that areas with a population density of 30,000 to 40,000 people per square kilometer are at lower risk compared to more densely populated regions [140]. The increase in population density often leads to the construction of substandard housing, inadequate water management systems and poor waste disposal practices, creating ideal conditions for the rise in disease cases in urban centers [139]. The primary vectors of dengue, i.e., *Ae. aegypti* and *Ae. albopictus* have adapted well to urban environments, with studies reporting that mosquito densities in urban settings are twice as high as those in rural areas [139]. Additionally, limited access to water and the creation of reservoirs for water storage during dry seasons, such as using uncovered containers, discarded plastic items or used tires precipitated by urban life, provides a conducive environment for the survival of *Aedes* mosquitoes alongside human populations [141]. Poor plumbing infrastructure and leaks from water pipes further contribute to stagnant water accumulation, which fosters mosquito growth and disease transmission [142].

Moreover, these mosquito species prefer habitats with moderate vegetation, including areas with constructed structures and medium-height trees. Urbanization not only creates such conducive habitats for them but also indirectly leads to climatic changes, which have recently emerged as another factor contributing to the increase in dengue cases [143,144].

**Implication for Iran:** Iran’s rapid urban growth, particularly the infrastructure deficit in southern cities, can be considered a key factor in the emergence of dengue fever in Iran. The synergy between unreliable water supply (necessitating storage) and inadequate waste management (providing containers) has created an urban ecology perfectly adapted to *Ae. aegypti*, transforming cities like Chabahar into high-risk transmission hubs [27,40,145].

#### 3.2.4. Global Travel and Trade

Increased international travel and trade has long been recognized as one of the most significant factors contributing to the global spread of dengue fever. Travelers play a crucial role in the worldwide epidemiology of dengue, as many acquire the infection while visiting endemic tropical regions but become ill only after returning home [142]. This continuous movement of infected humans allows dengue viruses and their vectors to spread to new areas, introducing novel virus serotypes where competent mosquito vectors are present [146,147]. The global trade of goods, particularly used tires and plants, can also transport *Aedes* mosquito eggs and larvae to different parts of the world and raise the prevalence of dengue [148]. Studies have found a positive correlation between the development of transportation networks, intraregional trade, migrant workers, religious pilgrimages, and infected tourists in the transmission of dengue virus and its vectors [142,149]. Phylogenetic analysis has also suggested that dengue virus in Saudi Arabia was likely introduced by migrant workers, religious pilgrims, or Saudi citizens traveling abroad [146]. Quarantine and controlled travel should always be seriously considered as deterrents to dengue fever and its vectors.

**Implication for Iran:** Iran’s geopolitical position makes it a net importer of dengue viruses. Constant introduction from endemic neighbors like Pakistan and Afghanistan via land borders and maritime trade through Chabahar port provides the repeated spark for local outbreaks in the receptive environment created by established vector populations [27,38,150].

#### 3.2.5. Awareness and Health Facilities

The awareness of dengue fever and the availability of health facilities are critical factors influencing the incidence and management of dengue outbreaks. The World Health Organization (WHO) emphasizes that effective dengue control requires a multisectoral approach, integrating health education and community participation [16]. Engaging communities in vector control activities and promoting awareness of dengue symptoms can significantly reduce transmission rates. For instance, targeted educational interventions have been shown to enhance community involvement in dengue prevention efforts, leading to better health outcomes [151]. A study conducted across five cities in Latin America highlighted the significant impact of community awareness on dengue transmission. The researchers revealed that while general knowledge about dengue and its vector was reasonably high, awareness regarding specific breeding sites, such as uncovered water containers and stagnant water, was notably inadequate. This lack of awareness was identified as a key factor contributing to the spread of dengue in these communities compared to other factors [152]. This finding underscores the importance of targeted educational campaigns aimed at increasing knowledge about the specific conditions that facilitate mosquito breeding. Effective communication through media and community outreach is essential to improve understanding of dengue transmission dynamics and preventive measures.

In addition to awareness, the capacity of health facilities plays a vital role in managing dengue outbreaks. Access to healthcare services, timely diagnosis, and appropriate treatment are crucial for reducing morbidity and mortality associated with dengue. Studies have shown that communities with well-equipped health facilities and trained personnel are better positioned to respond to dengue outbreaks effectively. Conversely, in areas where health facilities are lacking, the burden of dengue can be significantly higher due to delayed treatment and increased complications [153].

**Implication for Iran:** Iran’s susceptibility to dengue transmission is significantly compounded by synergistic gaps in public awareness and healthcare system preparedness [154,155]. Low community recognition of *Aedes* breeding behaviors—particularly in southern provinces with prevalent water storage practices—aligns with insufficient clinical awareness and diagnostic capacity among healthcare providers. This convergence creates a permissive environment for delayed detection and silent transmission during outbreaks, reflecting global patterns. The persistence of these dual deficiencies underscores the urgent need for targeted interventions that simultaneously address public education and clinical training gaps to mitigate dengue emergence in at-risk regions.

#### 3.2.6. Socioeconomic Factors

Socioeconomic factors are increasingly recognized as an important determinant of local dengue transmission, with the risk of disease exposure increasing as socioeconomic conditions worsen [77,156]. Some of the most significant socioeconomic factors influencing dengue fever include population density, housing type, sanitary conditions, lifestyle, economic status, occupation, and educational level [157,158]. Identifying these factors in communities is crucial for assessing disease epidemiology and implementing local interventions to address dengue risk factors.

Social inequality in communities is often associated with high human density, inadequate infrastructure, and poor environmental hygiene. These factors, along with various other factors such as poverty and lack of access to piped water and proper sewage systems, low public awareness, and uncontrolled urban expansion and slum development with high population density, which increase vector populations, are considered significant risk factors for dengue positivity [159,160,161]. Additionally, social contacts of symptomatic dengue cases are significantly associated with their mobility patterns in increasing disease cases in communities [162].

Lifestyle factors such as personal protection habits against mosquitoes, the amount of time spent outdoors, the way of resting in open spaces, or the use of air conditioning have been cited as influential factors in the occurrence of dengue fever, with the installation of air conditioning in homes leading to a reduced likelihood of dengue transmission [75]. Income and economic activities are also prominent socioeconomic factors associated with the epidemiological spread of dengue fever [163]. Some studies indicate a relationship between unemployment and high dengue incidence rates, as unemployment may lead to lifestyle behaviors that increase the risk of mosquito contact, such as spending more time inside and around the home during the day, resulting in increased vector exposure [164]. Populations with higher levels of education and employment are more likely to seek healthcare when infected with dengue, although these two factors alone cannot adequately explain the increase in reported dengue cases, as other factors such as individual awareness, proximity to hospitals and health centers, and more should also be considered. Some studies have found that urban areas with better employment and socioeconomic conditions have a higher risk of dengue infections, which requires more detailed studies in the future [158,165].

**Implication for Iran:** Socioeconomic factors play a fundamental role in dengue transmission dynamics. In endemic southern provinces, poverty is correlated with practices such as uncovered water storage and residence in areas with inadequate public sanitation, which directly increase vector exposure and impose a disproportionate disease burden on marginalized communities [145,158]. These structural inequalities pose additional challenges to the effective implementation of dengue control strategies in these regions.

### 3.3. Key Factors Contributing to the Prevalence of Dengue in Iran

Iran, located in Western Asia, is the seventeenth largest country in the world, with more than 80% of its territory classified as arid or semi-arid. Approximately 11.2% of Iran’s land area is used for agriculture, while forests, rangelands, deserts, and industrial/residential zones account for 8.7%, 52.1%, 19.7%, and 7.3% of the national landscape, respectively [166]. Iran is a vast country characterized by extreme geographical and climatic diversity. This includes arid and semi-arid central plateaus, high mountain ranges like Alborz and Zagros, dense subtropical forests in the north along the Caspian Sea, and long coastlines along the Persian Gulf and Oman Sea in the south with hot and humid conditions. This heterogeneity creates a wide range of ecological niches, some of which are highly conducive to the establishment of invasive *Aedes* mosquito vectors, i.e., *Ae. aegypti* and *Ae. albopictus*. A comprehensive review of available literature reveals that the prevalence of dengue fever in Iran exhibits a concerning upward trend, particularly from 2023 onwards, with local transmission becoming established in the southern provinces [27]. Despite this escalating threat, dedicated research remains limited and fragmented, often focusing broadly on dengue as a global health issue rather than addressing Iran-specific challenges [145]. As discussed in the global context (Section 3.2), the emergence and re-emergence of dengue are driven by a complex interplay of multifaceted factors. Of note, the epidemiology of dengue fever in Iran is likely influenced by a complex interplay of factors, consistent with the global mechanisms of dengue transmission discussed previously. However, it is important to emphasize that empirical evidence directly quantifying the effects of these specific factors on dengue incidence within Iran remains limited. Notwithstanding this research gap, several determinants have been posited in the literature as potential contributors to dengue dynamics in the Iranian context. These factors include: (A) Socio-cultural Context: Cultural and social contexts play a major role in shaping societal understanding and response to dengue outbreaks. In Iran, particularly in the southern regions, traditional beliefs and practices significantly influence how communities perceive and respond to infectious diseases [36]. For instance, in provinces such as Hormozgan and Sistan and Baluchestan, long-standing cultural practices of water storage—often in uncovered containers due to arid conditions and unreliable water infrastructure—create ideal breeding grounds for *Aedes* mosquitoes [145,167]. These practices, while culturally embedded, inadvertently facilitate dengue transmission. Pervasive misconceptions about dengue fever further complicate containment efforts. In many communities, the disease is misunderstood as a form of “seasonal fever”, leading to reliance on traditional remedies rather than seeking timely medical care. Such misconceptions, combined with low awareness of mosquito breeding habitats, hinder community participation in vector control programs [168,169]. Additionally, social behaviors such as outdoor gatherings and communal activities during times of high mosquito activity increase human-vector contact. Furthermore, resistance to modern interventions—such as insecticide spraying or removal of water containers—stems from deep-rooted beliefs and distrust of external authorities, further complicating public health efforts [155]. Therefore, the heterogeneity of cultural attitudes across different regions of Iran can impede public health efforts to reduce dengue cases and highlights the need for tailored, community-based interventions.

(B) Climate change is increasingly recognized as a critical determinant of vector-borne disease dynamics, and Iran is no exception. Long-term climatological records indicate that the country’s mean annual temperature has already increased by approximately 1.2–1.5 °C over the past five decades, with accelerated warming observed since the early 2000s (IPCC, 2021). Climate projection models—including CMIP6 simulations under Representative Concentration Pathways (RCPs) and Shared Socioeconomic Pathways (SSPs)—suggest that average surface temperatures in Iran could rise by 1.5–2.5 °C under moderate scenarios (RCP4.5/SSP2-4.5) and by up to 3.5–4.0 °C under high-emission scenarios (RCP8.5/SSP5-8.5) by 2050 [60]. National assessments further estimate an increase of up to 6.2 °C by 2100 under worst-case trajectories, with extreme heat days rising from 10 in 1990 to more than 200 annually by century’s end [166]. Given that over 80% of Iran’s landmass is classified as arid or semi-arid, even modest shifts in temperature and rainfall patterns have profound implications for disease ecology [166].

Regional studies highlight marked geographic heterogeneity. In the northern Caspian provinces of Gilan and Mazandaran, milder winters and more variable rainfall are projected, facilitating the persistence of *Ae. albopictus* and potentially enabling its year-round establishment [116]. Conversely, in the southern coastal provinces of Hormozgan and Sistan & Baluchestan—where recent dengue transmission has occurred—the climatic conditions are highly favorable for *Ae. aegypti* proliferation and sustained viral transmission. [166]. Chabahar (Sistan & Baluchestan) and coastal Hormozgan (including Bandar-Lengeh) experience long, hot, and humid summers with mild winters. In Bandar Lengeh, the mean annual temperature is approximately 27.6 °C, with summer daytime highs frequently exceeding 35 °C and winter minima falling near 14 °C. Relative humidity typically ranges from 64% to 67%, and total annual rainfall averages around 138 mm, concentrated in the cooler months (December–March) [170,171]. In Chabahar, the mean annual temperature is about 26.7 °C, with average summer highs reaching 35 °C and nighttime lows near 28 °C. Relative humidity remains elevated throughout the year (61–77%), and annual precipitation is roughly 110–120 mm, mostly during winter and early spring [170,171]. Despite limited rainfall, episodic precipitation creates short-lived collections of standing water that—combined with household water storage and inadequate drainage—provide abundant larval habitats that sustain *Aedes* populations even during the dry season.

Such environmental characteristics accelerate dengue virus replication and shorten the extrinsic incubation period (EIP) of the virus within *Ae. aegypti*, while high humidity enhances adult mosquito survival [172]. It is noteworthy that *Ae. albopictus* was first detected in Sistan & Baluchestan Province near the Pakistan border in 2009 and later in a coastal area close to Chabahar in 2013 [34]. However, subsequent intensive entomological surveillance failed to detect its establishment, suggesting that prevailing climatic conditions were not sufficiently suitable for its long-term persistence [173]. In contrast, this species successfully invaded Gilan Province in northern Iran in 2023 [116], emphasizing the strong influence of regional climate on vector distribution and establishment. Taken together, the prolonged warm seasons, recurrent heatwaves, and persistent coastal humidity in Hormozgan and Sistan & Baluchestan create an ecological niche highly conducive to *Ae. aegypti* survival and seasonal amplification of dengue transmission. Evidence from Tehran Province further demonstrates that semi-arid regions previously considered unsuitable could, under a 2 °C warming scenario, see seasonal windows of climatic suitability expand by up to 40%, significantly increasing the risk of vector establishment and dengue outbreaks in central Iran [61]. These findings collectively suggest that climate change will expand both the spatial and temporal windows of dengue transmission in Iran. By mid- to late-century, multiple provinces—including Gilan, Mazandaran, Tehran, Hormozgan, and Sistan & Baluchestan—may become climatically suitable for sustained transmission [62].

(C) Unplanned Urbanization and Habitat Creation: Iran has undergone one of the most rapid and sustained urban transitions globally. According to the United Nations Human Development Report, the urban population grew from 64.20% in 2000 to 75.94% in 2019 and is projected to reach 85.82% by 2050 [126]. This relentless urban expansion has been particularly pronounced in the southern coastal provinces of Sistan and Baluchestan, Hormozgan, and Bushehr—regions with confirmed populations of *Ae. aegypti* and persistent patterns of unplanned and haphazard development [27,68,174]. A critical consequence in these regions is the chronic inadequacy of municipal infrastructure, which fails to keep pace with demographic pressures. Intermittent piped water supply, a common feature in cities across these provinces, forces a high percentage of households to store water in a multitude of containers. These include underground cisterns (birke), above-ground tanks, and various drums, which constitute ideal breeding habitats for the highly domesticated *Ae. aegypti*. Concurrently, inefficient solid waste management systems lead to the widespread discarding of non-biodegradable containers, used tires, and plastic debris in peri-urban areas and vacant lots. These objects collect rainfall and irrigation runoff, creating a prolific and scattered network of larval breeding sites [145].

Furthermore, in northern provinces, recent years have witnessed significant land-use changes and accelerated villa construction. This development pattern has been associated with deforestation, increased local temperatures, and alterations to ecosystem dynamics and biodiversity [175]. These environmental modifications create favorable ecological conditions for the establishment and expansion of *Ae. albopictus* populations, which have been recently documented in northern regions [116].

The resulting urban environment in these provinces is a perfect ecological niche for invasive *Aedes* mosquitoes. The combination of water storage necessity and inadequate waste management directly links rapid, unplanned urbanization to the increased density of vector populations and a consequently higher risk of dengue transmission [145].

(D) Cross-Border and International Mobility: Iran’s position in global travel and trade networks represents a significant determinant of dengue introduction risk. The country shares extensive borders with dengue-endemic Pakistan and Afghanistan, facilitating cross-border movement of potentially infected individuals through both official and informal routes [16]. The strategic port of Chabahar receives substantial maritime traffic from dengue-endemic regions in South Asia and Africa, potentially enabling the introduction of both novel dengue virus serotypes and infected vectors through commercial goods such as used tires [38,150]. Internally, substantial travel from endemic southern provinces to major population centers such as Tehran and Isfahan—where competent vector species are not currently established—creates pathways for viral dissemination to new regions [27]. Religious tourism to holy cities (Mashhad and Qum) further compounds this risk by creating temporary transmission hotspots when viremic visitors encounter local mosquito populations.

(E, F) Ineffective Vector Control and Competing Disease Burden: Despite decades of experience in malaria control, Iran’s current vector control strategies demonstrate insufficient efficacy against dengue vectors [176]. The lack of sustainable larval source management and absence of effective community-based approaches substantially limit program effectiveness [22]. Furthermore, the emergence of insecticide resistance in Iranian *Ae. aegypti* populations significantly reduces the effectiveness of chemical control interventions [177]. Concurrently, Iran’s healthcare system faces competing priorities from persistent communicable diseases including malaria and leishmaniasis, coupled with a growing burden of non-communicable diseases [169]. This diversion of resources and attention diminishes dengue’s prioritization within the national health agenda, consequently constraining surveillance capabilities, funding allocation, and targeted interventions [150,169].

(G) Vaccine Availability: The absence of a safe and universally accessible dengue vaccine in Iran remains a critical barrier to prevention. Although global advances have produced candidate vaccines, none have been integrated into Iran’s national immunization programs, largely due to concerns regarding efficacy, cost, and serotype-specific performance. This gap leaves vector control and case management as the primary tools for reducing disease burden [178].

(H) Deficiencies in Healthcare Provider Knowledge and Practices: Studies conducted in Iran have revealed significant deficiencies in knowledge, attitudes, and practices (KAP) among healthcare providers regarding dengue fever diagnosis, management, and prevention. Research among healthcare workers in northern Iran demonstrated substantial gaps in knowledge related to dengue symptoms, transmission mechanisms, and control measures [155]. This is particularly concerning in Sistan and Baluchestan Province (Chabahar County), where active dengue transmission occurs. In this outbreak-prone region, Najati et al. [154] reported that a substantial proportion of participants lacked formal training in dengue management and fewer than half recognized the importance of larval source reduction in vector population control. Consequently, this lack of awareness and training among healthcare providers may lead to delayed diagnosis and inefficient case management of dengue fever, thereby increasing the risk of disease outbreaks and secondary transmission in vulnerable communities. These systematic KAP limitations represent a critical vulnerability in Iran’s healthcare system’s ability to respond effectively to emerging arboviral diseases.

(I) Blood Safety and Transfusion Risk: The risk of transfusion-transmitted dengue (TTD) is an emerging concern for Iran’s blood safety system. A seroepidemiological study of blood donors in Chabahar found a dengue IgG seroprevalence of 4.8%, providing clear evidence of community exposure and a potential reservoir of asymptomatic, viremic donors [37]. Despite this evidence, the Iranian Blood Transfusion Organization (IBTO) does not implement routine nucleic acid testing (NAT) or antigen screening for dengue virus in blood donations. This policy gap is primarily due to the high cost of universal screening and the disease’s nascent status. However, as local transmission intensifies, the probability of TTD increases, necessitating a formal risk assessment and the consideration of targeted or seasonal screening strategies in endemic provinces to safeguard the blood supply.

(J) Public Health Infrastructure and Historical Focus: Iran’s public health infrastructure has been historically shaped by and optimized for combating specific endemic diseases, notably malaria and leishmaniasis [179]. The resulting strategies, expertise, and institutional memory are consequently deeply ingrained in controlling the vectors associated with these specific diseases (e.g., *Anopheles* mosquitoes for malaria). As a result, the existing surveillance systems, vector control units, and clinical guidelines are not yet fully adapted to the distinct epidemiological and entomological challenges posed by dengue fever and its primary vector, *Ae. aegypti*. This institutional inertia represents a significant vulnerability that could potentially facilitate more extensive dengue transmission during outbreaks in Iran. This mounting threat necessitates a significant paradigm shift—from an exclusive focus on rural *Anopheles* control to an integrated approach that simultaneously addresses both urban *Aedes* management and sustained malaria prevention; from compartmentalized, disease-specific approaches to a holistic vector-borne disease strategy; and from a reactive outbreak response model to proactive, data-driven surveillance [179].

It should be noted that these factors collectively underscore the urgent need for targeted interventions and policy adjustments to mitigate the growing threat of dengue fever in Iran (Section 3.3.1).

While the aforementioned factors may contribute to dengue emergence in Iran, it is noteworthy that the majority of autochthonous transmission have been concentrated in Chabahar County, Sistan and Baluchestan Province, despite established populations of *Ae. aegypti* in other similar districts. Surprisingly, no comprehensive studies have investigated the underlying drivers of this localized surge. Field observations by the authors and personal communications with Iran’s CDC suggest three potential determinants: (1) Rapid expansion of informal settlements (urban fringe development), (2) Increased commercial traffic through Chabahar Port, and (3) Political changes in neighboring Afghanistan facilitating imported case influx and subsequent surge in local transmission. This epidemiological pattern underscores the critical need for targeted original research examining these unique regional dynamics.

#### 3.3.1. Targeted Control Recommendations for Dengue Fever in Iran

Based on the analysis of targeted control recommendations derived from the key factors influencing the emergence of dengue fever in Iran, specific interventions are prioritized below to guide national policies and inform health policymakers for reducing dengue incidence in expanding risk areas. This prioritization is designed to maximize public health impact by simultaneously addressing the synergistic drivers identified in our synthesis, while establishing sustainable, long-term control capacity.

1. Enhance Community Engagement and Public Awareness: Given the unique socio-cultural context influencing perceptions around dengue, culturally sensitive health education campaigns should be designed and implemented to address local beliefs and behaviors through educational -strategies such as Communication for Behavioral Impact (COMBI). Partnering with community leaders and using media tailored to diverse populations can increase awareness to translate education into action.
2. Implement Urban Planning Interventions to Manage Mosquito Breeding Sites: Urbanization-induced habitat expansion demands incorporation of vector habitat reduction into urban infrastructure development and waste management policies. Installing proper drainage, regulating water storage, and improving sanitation in rapidly expanding urban, peri-urban and slam areas can reduce suitable *Aedes* breeding sites significantly.
3. Adopt and Scale-Up Integrated Vector Management (IVM): Current vector control shortcomings necessitate a comprehensive IVM approach combining chemical, biological, and environmental methods with community participation. This integrated framework could include the use of biological larvicides, and sustainable environmental practices, alongside careful management of insecticide resistance, to ensure more effective and sustainable vector suppression. Moreover, while *Aedes* mosquitoes primarily bite during the day, the use of insecticide-treated nets (ITNs) could help protect hospitalized dengue patients in healthcare settings and vulnerable groups who exhibit daytime resting behaviors.
4. Strengthen Public Health Infrastructure and Multi-Sectoral Coordination: Iran’s public health infrastructure needs strategic upgrades including enhanced laboratory capacity, data management systems, retraining personnel, and revising national policies to address this new threat. In addition, stronger inter-sectoral coordination among health, environment, agriculture, urban development, and education sectors is essential to create a resilient and adaptive dengue control framework.
5. Enhance Healthcare Professionals’ Training and Capacity: Regular training programs to improve healthcare workers’ knowledge, diagnostic skills, and management of dengue cases are needed to enhance clinical outcomes and outbreak response efficiency. Strengthening guidelines and providing resources will support standardized care and effective education of patients and communities.
6. Strengthen Cross-Border Surveillance and International Collaboration: Given Iran’s proximity to dengue-endemic countries and increasing international travel, enhancing cross-border cooperation through data-sharing platforms and joint vector surveillance efforts is critical. Screening protocols at ports of entry and coordination with neighboring public health authorities can reduce viral importation and contain local transmission.
7. Combat Misconceptions and Build Trust in Health Authorities: Active community engagement strategies, transparency in communications, and involvement of trusted local figures are essential to overcome misconceptions and foster community participation in dengue prevention and control. Establishing feedback mechanisms can improve responsiveness and trust, thereby enhancing compliance with control measures.
8. Predictive modeling and climate monitoring systems must be integrated into public health planning to anticipate shifts in *Aedes* mosquito distribution and seasonality due to progressive warming. Early warning systems based on climatic data can inform timely vector control responses and resource allocation in at-risk regions previously unaffected by dengue.
9. Prioritize Dengue in the Context of Competing Disease Burdens: Policy makers should ensure dengue fever receives adequate attention and funding within Iran’s public health agenda despite existing burdens from other communicable and non-communicable diseases. Cross-program synergies and integrated disease surveillance systems can optimize resource utilization and response capabilities.
10. Improve Blood Safety and Donor Screening Practices: Robust screening protocols for blood donations must be implemented and regularly audited to prevent transfusion-transmitted dengue, particularly in endemic or outbreak settings. Investment in sensitive diagnostic assays and staff training are essential components of safe blood supply management.
11. Expand Dengue Vaccination Access and Research: Addressing vaccine availability barriers through national immunization program integration and advocacy for equitable access to newly developed safe, tetravalent dengue vaccines is critical when and where advisable. Simultaneously, promoting surveillance to monitor vaccination outcomes and vaccine effectiveness will guide optimal immunization strategies.

#### 3.3.2. Current Policies for Dengue Surveillance and Control in Iran

Iran’s current national policy for dengue fever control, is delineated in the national protocols for vector surveillance and control [22], and case detection and management [27]. Vector control surveillance is a dynamic and scenario-based strategy centered on Integrated Vector Management (IVM). Recognizing the significant threat posed by the invasive *Ae. aegypti* and *Ae. albopictus* mosquitoes, Iran has adopted a tiered response framework built upon three distinct epidemiological scenarios. Each province or even sub-region within a province is classified into one of these scenarios, which dictates the specific set of surveillance and control interventions to be implemented.

In Scenario I, where the vector is not established but there is a high risk of entry through points of entry like seaports and airports, the policy focuses on preventive entomological surveillance using tools like ovitraps, rigorous environmental management to eliminate potential larval habitats, and public education. Concurrently, human surveillance is strategically targeted at points of entry and within the healthcare system. Syndromic surveillance is implemented to identify suspected cases among travelers and individuals with a history of travel to endemic countries. The primary objective is the rapid detection and management of imported cases to prevent the introduction of the virus into local populations. Should a vector be detected, the immediate goal is its rapid elimination.

In Scenario II, which is characterized by limited establishment in a defined area (<25 km^2^), the strategy escalates to an intensive containment and elimination effort. This includes enhanced mapping of the infested areas, targeted adulticide interventions via space spraying (fogging), weekly larviciding, and intensified source reduction. At this stage, human surveillance is intensified and becomes highly targeted. Syndromic surveillance is reinforced in the affected areas, complemented by active case finding and community screening within a 500-m radius of mosquito collection points. The use of Rapid Diagnostic Tests (RDTs) is scaled up for prompt case detection and management.

Finally, for Scenario III, where widespread establishment (>25 km^2^) makes vector elimination unfeasible, the policy objective shifts to sustainable population management through routine IVM. This involves continuous larval source reduction, regular larviciding, focal adulticiding, and close monitoring of vector indices and insecticide resistance, all aimed at reducing the vectorial capacity to prevent local transmission, especially from imported cases. In this scenario, human surveillance becomes a cornerstone of the public health response, fully integrated with entomological activities. A robust, community-wide syndromic surveillance system is activated across all healthcare levels. Widespread testing of suspected cases, intensive case detection, and seroprevalence studies are implemented to monitor transmission dynamics. The mandatory immediate reporting (within less than 24 h) of any probable or confirmed dengue case enables rapid vector control around patients’ homes and workplaces. Public awareness campaigns are intensified to promote personal protection and healthcare-seeking behavior, forming a critical barrier against sustained local transmission.

## 4. Discussion

Dengue fever, a mosquito-borne viral infection, has seen a significant rise in incidence globally over the past few decades. According to the Centers for Disease Control and Prevention (CDC), as of the beginning of 2024, the number of dengue positive cases is twice the total number of cases reported during 2023. The Southern Hemisphere exhibited significant disparities in the epidemiological burden of dengue fever. Surveillance data indicated approximately 11.56 million confirmed cases and 7175 dengue-associated deaths in southern latitudes—reflecting a 4.5-fold higher case burden and three times greater mortality compared to the Northern Hemisphere, which recorded 2.57 million infections and 2333 deaths [180]. Brazil has documented the highest number of cases in 2024, with over 9 million, followed by Argentina, Paraguay, Peru, and Colombia [20]. In 2025, dengue fever continues to pose a significant public health challenge in the region, with Brazil alone confirming 2,788,443 cases as of June 26, and the total dengue cases across affected countries approaching three million, accompanied by over 1400 dengue-related deaths [181]. In March of 2024, health officials in Puerto Rico and US Virgin Islands declared an outbreak of dengue, with transmission persisting through 2025. Additionally, local cases of dengue have been reported in three U.S. states: Florida, California, and Texas, with Florida experiencing ongoing local transmission during 2025 [182]. Higher than normal numbers of dengue cases are reported by countries in Table 5 [182].

In mainland Europe, France and Italy reported 21 and 1 autochthonous cases of dengue in July and August 2024, respectively, although no locally acquired cases have been recorded in 2025. However, dengue infections have been identified in the outermost regions of the European Union [20]. In South-East Asia, Bangladesh reported an increase in the total number of dengue cases in 2025 compared to the same period in 2024, according to the country report published on 25 May 2025 [20]. Meanwhile, in the Eastern Mediterranean Region (EMRO), dengue has emerged as a critical public health concern in Iran, since 2008, when the first imported case was identified in a 58-year-old woman with a history of travel to Malaysia [155]. Subsequently, Iran reported an average of 20 imported dengue cases annually between 2017 and 2023. However, there was a notable increase in imported dengue cases in 2024, with 140 cases reported between 1 April and 30 August, which probably led to the local transmission of the virus in Iran (Table 1) [183]. On 14 June 2024, the Ministry of Health and Medical Education (MoHME) of Iran reported the first two locally acquired cases of dengue in Bandar-Lengeh, Hormozgan Province, southern Iran [183]. The total number of autochthonous dengue cases in the country has since risen, with reported cases from Bandar-Lengeh and Chabahar Counties in Sistan and Baluchistan Province, southern Iran [27]. This is an important warning, raising concerns about the potential for a disease outbreak across the country owing to the spread of its competent vectors in the south and north of the country. Concurrently, various vector control initiatives aimed at reducing mosquito populations—through methods such as larval source management, insecticide spraying, and public health awareness campaigns—are being conducted [22]. Nevertheless, these interventions face considerable challenges, including poor environmental sanitation, widespread insecticide resistance, and the high costs associated with sustainable implementation of the control measures. These challenges underscore the urgent need to comprehend the multifaceted factors contributing to the emergence of such diseases. Understanding these factors is essential for developing effective prevention and control strategies.

Identifying factors affecting the emergence and re-emergence of dengue has become a hot topic in current research [184]. This review systematically identified eleven interconnected factors driving dengue emergence in Iran, which collectively answer the core research question regarding causative elements and control strategies. The convergence of socio-cultural practices (A), particularly water storage in uncovered containers, with unplanned urbanization (C) and unreliable water infrastructure creates an ideal ecological niche for *Ae. aegypti* propagation [40,145]. This established vector population is continuously replenished through cross-border mobility (D) from endemic neighboring countries, while climate change (B) progressively expands the vectors’ suitable geographical range into new regions such as central and northern provinces [60,116]. The situation is critically exacerbated by systemic vulnerabilities, including gaps in vector control programs (E), competing disease burdens (F), limited vaccine availability (G), healthcare provider knowledge deficits (H), blood safety concerns (I), and a public health infrastructure (J) historically optimized for other disease priorities [154,155,177]. This complex interplay demonstrates that Iran’s dengue emergence cannot be attributed to isolated factors but rather results from their synergistic interactions, where unplanned urbanization intensifies water storage problems, climate change enables vector expansion, and healthcare system limitations amplify transmission potential.

The implications for Iran, as detailed in the results, are important and demand a paradigm shift in public health strategy. The projected expansion of climatically suitable areas for *Ae. albopictus* into central and northern provinces, including Tehran, necessitates a pre-emptive expansion of surveillance beyond the current southern foci [62,154]. The specific Iranian context, where transmission in arid southern provinces is primarily driven by human water storage practices rather than rainfall, highlights that control strategies must prioritize improving municipal water infrastructure and regulating water storage as a primary intervention [22,68]. Furthermore, the role of international travel and trade through hubs like Chabahar Port as a conduit for viral introduction underscores the critical need for enhanced cross-border surveillance and port security measures [27,150]. The deficiencies identified in healthcare worker knowledge and the competing burdens of other communicable diseases reveal an urgent need to strengthen clinical diagnosis, case management, and outbreak response capacity within the Iranian health system [154,169].

When considering control strategies for Iran, several approaches emerge as particularly relevant. Community-based interventions that address water storage practices through culturally appropriate education campaigns could significantly reduce breeding sites in southern provinces [151]. Environmental management targeting artificial containers in peri-urban and urban areas should be prioritized over insecticide-based approaches, especially given the emerging resistance reported in Iranian *Ae. aegypti* populations [22,177]. The establishment of an early warning system that incorporates climatic, entomological, and epidemiological data could provide crucial lead time for outbreak response, particularly in the expanding risk areas of central and northern Iran [41]. Strengthening the healthcare system’s capacity for dengue diagnosis and management is equally crucial, especially in the underserved provinces where the burden is currently concentrated [154,169].

In the global context, synergistic interactions among the identified factors were similarly observed. For instance, Nakasa et al. documented that the ratio of areas with high and very high population density, accompanied by elevated climate suitability for dengue virus (DENV) transmission, increased from 58.5% to 66.2% and from 48.1% to 63.6%, respectively. This finding highlights the significant interplay between climate factors and population growth in contributing to the rising incidence of dengue fever [185]. Akinsulie and Idris showed that outbreaks of dengue in France and Italy are attributed to the increasing impact of global warming and international travel. Not to mention, the combination of these factors makes the countries more susceptible to dengue outbreaks, even in regions that have not traditionally experienced the disease [186]. In 2024, Southeast Asian regions, particularly Bangladesh, Nepal, and India, reported an increase in dengue cases compared to the same period of 2023. Various factors, including population displacement, unplanned urbanization, increased human connectivity, and climate change, especially the impact of monsoon rains have played a critical role in the heightened disease burden in the region [187]. It is essential to state that the importance of monsoons in the emergence of dengue fever has become increasingly evident in recent years, particularly due to the interplay of climate change and shifting weather patterns. Monsoons, traditionally linked with increased mosquito breeding due to stagnant water, have intensified in many regions, leading to unprecedented outbreaks. For instance, during the 2023 monsoon season in Bangladesh, rainfall levels were reported to be 13% above the long-term average, contributing to a surge in dengue cases and marking one of the deadliest outbreaks recorded [107]. Research indicates that each additional rainy day can increase dengue cases by approximately 6% in the following month, highlighting the direct correlation between monsoon rainfall and disease incidence [188].

According to researchers attributing the rise in dengue fever to climate change, the disease is expected to impact 60 percent of the global population by the year 2080 [189]. In Iran, despite the country’s high susceptibility to climate-sensitive diseases and its vulnerability to the effects of climate change, such as rising temperatures and increased frequency of extreme weather events, the issue of climate change and health is not yet a priority within the national health system. This critical gap necessitates immediate and dedicated attention at the highest levels of health policy-making and senior management to allocate sufficient resources and foster robust multi-sectoral collaboration [166]. Therefore, addressing these factors through integrated management strategies is essential for reducing the impact of dengue fever globally. By targeting these factors, international collaborations, and implementing evidence-based interventions, the burden of dengue fever can be managed worldwide [190]. For instance, by effectively reducing global warming, a significant reduction in the number of dengue cases can be achieved. The Paris Agreement sets a goal of limiting the increase in global average temperature to 1.5 degrees Celsius above pre-industrial levels. Consequently, it is projected that the number of dengue cases could decline by 300,000 per year by 2050 and by 500,000 per year by 2100 [42]. Additionally, by curbing global warming, the spread of dengue to regions with lower incidence rates could be prevented. This underscores the critical importance of global cooperation and national commitment to climate change mitigation. For countries like Iran, this means not only participating in international efforts but also developing robust national surveillance systems, early warning mechanisms, and public health preparedness plans specifically designed to address climate-sensitive diseases like dengue fever.

To address the impact of above-mentioned factors on the emergence and re-emergence of dengue, the following strategies can also be recommended:

- Improving early warning systems based on climate data to predict and prepare for potential outbreaks
- Promoting climate-resilient urban planning and infrastructure to minimize the creation of mosquito breeding habitats
- Promoting sustainable land use practices and limiting deforestation
- Improving urban planning and waste management to minimize mosquito breeding sites
- Enhancing disease surveillance and outbreak response capabilities
- Implementing travel-related interventions, such as screening and quarantine measures, to limit the importation of dengue cases
- Improving access to healthcare services and strengthening public health infrastructure
- Promoting intra- and inter-sectoral collaborations to address the factors affecting the spread of dengue and its vectors
- Promoting and enforcing international treaties to tackle the vulnerabilities conducive to dengue spread
- Advocacy both nationally and internationally through responsible relevant agencies to mitigate and manage the impact of factors causing the spread of dengue
- Conducting educational campaigns to raise awareness about dengue among healthcare workers and the general population
- Providing training and resources to healthcare facilities for the diagnosis and management of dengue cases
- Engaging communities in vector control activities and promoting preventive measures, such as eliminating standing water and maintaining a clean environment, and using personal protective measures.

The multi-pronged strategies proposed are supported by robust evidence from their successful implementation in diverse international settings, where they have contributed significantly to reducing the burden of dengue fever. For instance, the strategy of improving early warning systems based on climate data has been operationalized in countries like Brazil, where climate-informed forecasting models have proven valuable in extending the lead time for public health interventions and improving outbreak preparedness [191]. Similarly, in Sri Lanka, the establishment of an ICT-based real-time dengue surveillance system has markedly enhanced early outbreak detection and facilitated timely public health responses, thereby mitigating both the spread and impact of the disease [192]. Furthermore, the effectiveness of community engagement in vector control and environmental management was conclusively demonstrated in the landmark “Camino Verde, the Green Way” cluster-randomized controlled trial conducted in Mexico and Nicaragua. This community-based intervention, which focused on eliminating mosquito breeding sites, led to a remarkable reduction in dengue incidence in the intervention communities, providing high-level evidence for this approach [193].

Furthermore, Singapore’s national dengue control program exemplifies the success of climate-resilient urban planning and integrated vector management—characterized by a combination of strict environmental enforcement, targeted vector control, and continuous public education. The country’s long-standing, evidence-based strategy integrates urban infrastructure design—including optimized drainage systems, regulated construction practices, and proactive environmental management—with rigorous entomological surveillance, targeted vector control, and sustained public education [194,195,196]. These coordinated efforts, embodied in initiatives such as the nationwide “5-Step Mozzie Wipeout” campaign [195], have contributed to maintaining low dengue endemicity despite environmental conditions highly conducive to *Aedes* proliferation. These examples from Sri Lanka, Latin America, and Singapore validate that the core strategies of climate-informed surveillance, community mobilization, and integrated vector management are not only theoretically sound but have been proven effective in diverse epidemiological and cultural contexts, providing a strong evidence base for their recommended adoption.

It is worth mentioning that in recent decades, innovative technologies have increasingly transformed the landscape of dengue fever surveillance and control, offering promising new strategies to complement traditional methods. These novel approaches harness advancements in remote sensing, artificial intelligence (AI), genetic engineering, wolbachia, sterile male techniques, new traps, and vaccine development to enhance the precision, efficiency, and sustainability of dengue management. Remote sensing techniques enable high-resolution mapping of *Aedes* mosquito habitats and environmental risk factors, facilitating targeted vector control interventions by identifying breeding sites that may not be easily accessible through conventional ground surveys [197]. Concurrently, AI and machine learning models are emerging as powerful tools for outbreak prediction, integrating complex climatic, entomological, and epidemiological datasets to generate timely forecasts that can guide proactive public health responses [198]. Furthermore, ongoing progress in the development and deployment of next-generation dengue vaccines—including tetravalent and live-attenuated candidates—holds promise for expanding immunization coverage and enhancing long-term disease prevention. However, challenges in vaccine safety, efficacy, delivery, and accessibility remain and must be addressed to maximize their impact on public health [199]. Collectively, these cutting-edge technologies represent a paradigm shift toward more integrated, data-driven, and adaptive approaches that are essential to effectively shaping the future of dengue control in endemic and emerging areas alike.

## 5. Conclusions

This narrative review has elucidated the multifaceted factors driving the emergence and re-emergence of dengue fever in the 21st century. The findings underscore the intricate interplay between climate change, environmental alterations, unplanned urbanization, and global mobility in shaping the transmission dynamics of this mosquito-borne disease. This review provides valuable insights for researchers and policy makers, suggesting that a comprehensive approach combining vector control and education with the participation of local, national and international communities holds promise for lower prevalence and improved public health in an interconnected world in the near future. Addressing the factors enlisted in this review by national and international responsible agencies/organizations is an urgent need to bring to halt and possibly reverse the chain of events leading to increase in the incidence and occurrence of dengue worldwide. The recent surge in local dengue fever cases in Iran has become alarming for its further global spread. Therefore, global partnerships, including multinational research and development collaborations and international funding mechanisms, are essential to support regional and Iran’s efforts in strengthening surveillance, improving vector control strategies, and advancing capacity building. Such collaborations facilitate knowledge transfer, access to advanced technologies, and mobilization of critical resources necessary to effectively address this public health challenge, thereby substantially contributing to the reduction in the global burden of dengue. Furthermore, enhanced interdisciplinary cooperation among public health, health education, urban planning, climate science, and environmental health sectors is also required to develop comprehensive and sustainable solutions to the dengue crisis.

## Figures and Tables

**Table 1:** Monthly cumulative frequency of dengue cases in Iran (April 2024–mid-2025).

Year	Month	Total Cases	Local Cases	Imported Cases	City		Travel History					
					Chabahar	Bandar Lengeh	United Arab Emirates	Pakistan	Afghanistan	Oman	Turkey	Benin
2024	April	-	-	-	-	-	-	-	-	-	-	-
	May	-	-	-	-	-	-	-	-	-	-	-
	June	111(111) **	5(5)	106(106)	-	5(5)	103(103)	1(1)	-	1(1)	0	1(1)
	July	151(40)	12(7)	139(33)	1(1)	11(6)	130(27)	7(6)	-	1(0)	0	1(0)
	August	161(10)	21(9)	140(1)	10(9)	11(0)	131(1)	7(6)	-	1(0)	0	1(0)
	September	189(28)	44(23)	-	33(23)	11(0)	-	-	-	-	-	-
	October	221(32)	71(27)	-	60(27)	11(0)	-	-	-	-	-	-
	November	303(82)	141(70)	-	130(70)	11(0)	-	-	-	-	-	-
	December	875(572)	677(536)	-	666(536)	11(0)	-	-	-	-	-	-
	January	1076(201)	865(188)	-	852(186)	13(2)	-	-	-	-	-	-
	February	1106(30)	891(26)	-	878(26)	13(0)	-	-	-	-	-	-
	March	1127(20)	922(32)	205(65)	897(19)	25(12)	132(1)	69(62)	1	1(0)	1(1)	1(0)
2025	April	6(6)	6(6)	-	6(6)	-	-	-	-	-	-	-
	May	37(31)	37(31)	-	37(31)	-	-	-	-	-	-	-
	June	188(151)	187(150)	1(1)	187(150)	-	-	1(1)	-	-	-	-
	July	278(90)	271(84) *	7(6)	271(84)	-	-	7(6)	-	-	-	-

* Two cases were reported in Zahedan and two cases in Iranshahr, with all four patients having a history of travel to Chabahar; ** The numbers in parentheses indicate the raw frequencies.

**Table 5:** Countries with higher-than-normal numbers of dengue cases from 2024 up to July 17, 2025.

	Americas		Africa	Eastern Mediterranean	South-East Asia	Western Pacific
2024	Colombia	Guatemala	Burkina Faso	Afghanistan	India	French Polynesia
	Costa Rica	Guyana	Cape Verde	Iran		
	Cuba	Honduras	Central African Republic	Pakistan		
	Ecuador	Mexico	Ethiopia			
	El Salvador	Panama	Ghana			
	Dominican Republic	Saint Lucia	Mali			
	Grenada	Trinidad and Tobago	Sudan			
			Togo			
2025	Colombia		Comoros	-	Bangladesh	Cook Islands
	Ecuador		Mali			Fiji
	Guatemala		Sudan			French Polynesia
	Panama					Kiribati
						Philippines
						Samoa
						Tonga
						Tuvalu

## Data Availability

No new data were created or analyzed in this study. Data sharing is not applicable to this article.

## Appendix A. Sample Search Strategy (PubMed)

((“Dengue fever”[Title/Abstract]) OR (“Dengue virus”[Title/Abstract]) OR (Dengue[Title/Abstract])) AND ((emergence[Title/Abstract]) OR (re-emergence[Title/Abstract]) OR (outbreak[Title/Abstract]) OR (epidemiology[Title/Abstract]) OR (“risk factors”[Title/Abstract])) AND ((“*Aedes aegypti*”[Title/Abstract]) OR (“*Aedes albopictus*”[Title/Abstract])) AND (Iran[Title/Abstract])).
